# A novel mutation in TRPV3 gene causes atypical familial Olmsted syndrome

**DOI:** 10.1038/srep21815

**Published:** 2016-02-23

**Authors:** Cheng Ni, Ming Yan, Jia Zhang, Ruhong Cheng, Jianying Liang, Dan Deng, Zhen Wang, Ming Li, Zhirong Yao

**Affiliations:** 1 Department of Dermatology, Xinhua Hospital, Shanghai Jiaotong University School of Medicine, 1665 Kongjiang Road, Shanghai 200092, China

## Abstract

Olmsted syndrome (OS) is a rare keratinization disorder, typically characterized by two primary diagnostic hallmarks—mutilating palmoplanter and periorificial keratoderma. However, there’s a growing body of literature reporting on the phenotypic diversity of OS, including the absence of aforementioned hallmarks and the presence of some unusual clinical features. Here we presented an atypical familial case of OS that could be confused with Huriez syndrome due to the presence of a scleodactyly-like appearance and tapered fingers in the proband. We ruled out this possibility and made a definitive diagnosis of OS based on clinical features and a genetic assay. Recently, mutations in *TRPV3* associated with autosomal dominant or recessive OS continued to be reported, thus conducing to clarifying the underlying relationship between the genotype and phenotype of OS. So we further explored the genotype-phenotype correlation by integrating functionl assays with *in silico* predictions. Our research not only redefined the phenotypic spectrum of OS, but also provided concrete molecular insights into how mutations in a single gene can lead to significant differences in the severity of this rare disease.

Olmsted syndrome is a rare genodermatosis featuring bilateral progressive mutilating palmoplanter keratoderma (PPK) and periorificial keratotic plaque^1^. It has generally been accepted that the two above-mentioned clinical manifestations were prerequisites for the diagnosis of this disease^2^. As a result, some researchers have suggested that atypical cases without the classic combination of clinical signs may not really belong to OS^3^,^4^. Meanwhile, variable clinical features associated with OS have been continually described, highlighting the phenotypic diversity of OS. Rare cutaneous anomalies such as nail dystrophy, leukokeratosis of oral mucosa, hyperhidrosis or hypohidrosis of the palmoplantar, can also be associated^5^,^6^. Systemic complications relevant to OS including congenital deafness, mental retardation, osteoporosis, squamous cell carcinoma and malignant melanoma, have also been reported^7^.

Nonspecific histopathological and ultrastructural findings and the clinical overlap with other keratinization disorders cause difficulty in making a definite diagnosis^8^, whereas, genetic assay offers the best route to an accurate diagnosis owing to the identification of pathogenic loci of OS. Recently, whole-exome sequencing was used to detect pathogenic gain-of-function mutations in *TRPV3*, which encodes a transient receptor potential vanilloid cation channel, in autosomal-dominant OS^9^. Subsequently, a homozygous mutation and a compound heterozygous mutation were also implicated in autosomal-recessive OS^10^,^11^. Although mutations in *MBTPS2* have additionally been shown to be associated with X-linked recessive OS^12^, the concomitance of OS-like features in a patient with ichthyosis follicularis atrichia and photophobia (IFAP) syndrome suggests the X-linked OS may not be an independent entity^13^.

In the present research, we recruited an atypical familial case of OS in which patients did not present with periorificial keratoderma and alopecia. Additionally, some clinical signs in the proband, such as cone-shaped fingers and a scleodactyly-like appearance, overlapped with symptoms previously described in Huriez syndrome. Initially, the characteristic lesion of the proband’s plantar was examined histopathologically and ultrastructurally. Meanwhile, due to the occurrence of pseudoainhum in the present case, we screened five genes (*KRT1*, *GJB2*, *SLURP1*, *LOR* and *TRPV3*), which were previously indicated in the pathogenesis of genodermatosis with mutilating palmoplantar keratoderma^6^,^9^. We identified the causative mutation and dissected the structural changes of the protein by molecular modeling.

We also conducted a further study, intergrating the i*n silico* and *in vivto* assay data, to correlate the phenotypic spectrum of OS with pathogenic mutations.

## Results

### Clinical data

**Figure 1:**
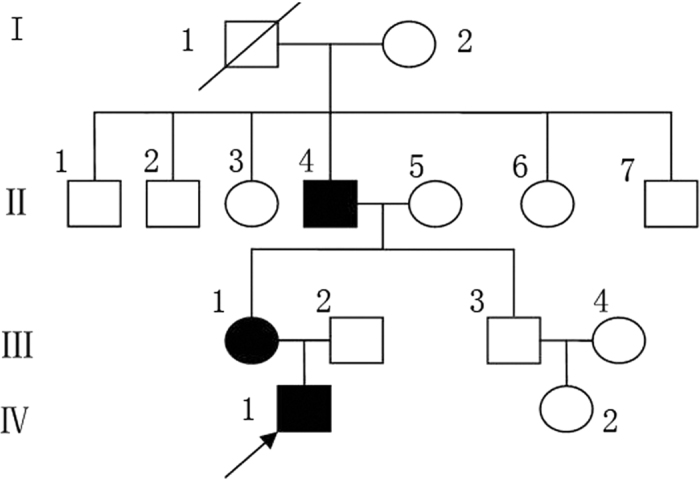
Pedigree of the OS family. The arrow refers to the proband.

**Figure 2:**
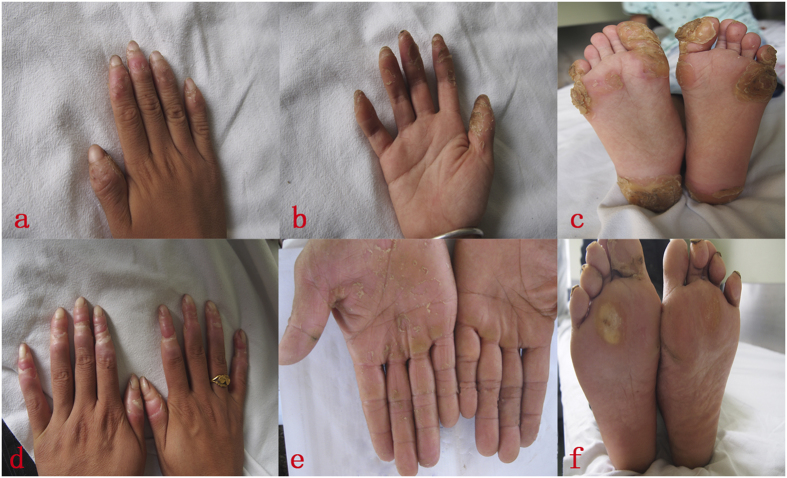
Clinical presentation of three patients in the Chinese family. The proband (IV1) showed symmetric, focal palmoplantar keratoderma, scleodactyly-like appearance, cone-shaped fingers, desquamation and warmth in the extremities. The hyperkeratotic plaques remained focal all through and primarily distributed in islands on the pressure sites, with no transgrediens extension (**a–c**). His mother (III1) presented with focal palmoplantar keratoderma and obvious pseudoainhum (**d**). His maternal grandfather (II4) manifested with focal keratotic plaques on the soles and desquamation on the palms (**e,f**).

The pedigree in this study included three affected individuals from a three-generation Chinese family (Fig. 1). The proband was a six-year-old boy, referring to our outpatient department with complaints of symmetric, focal PPK, acute flares of warm-elicited pain and itching, a scleodactyly-like appearance, cone-shaped fingers, mild pseudoainhum, and desquamation in the extremities. The hyperkeratotic plaques remained focal all through and primarily distributed in islands on the pressure sites, with no transgredient extension. There was neither periorificial hyperkeratosis nor the anomalies of hairs and nails. Intriguingly, the symptoms of the palm were much milder than those of the plantar in the proband. He presented with mild keratosis and peeling on the palms, and thick yellow-brown, fissure hyperkeratic plaques on the pressure sites of the plantar (Fig. 2a–c). Systemic examination revealed no abnormalities. Growth and mental developments were appropriate for his age. The parents were allegedly nonconsanguineous. Other two patients in this family was the proband’s mother and his maternal grandfather (Fig. 1). His mother’s clinical signs were focal PPK with obvious pseudoainhum (Fig. 2d), while his maternal grandfather manifested with focal keratotic plaques on the soles and desquamation on the palms (Fig. 2e,f). However, symptoms such as: acral hyperalgesia, severe itching, or warmth in the extremities, were absent in the two patients.

### Pathology characteristics

**Figure 3:**
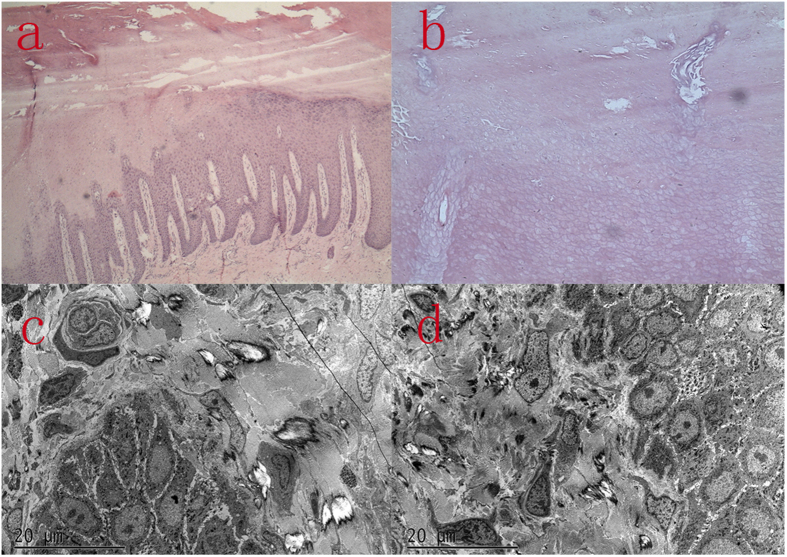
Histopathologic and ultrastructural features of the proband’s skin lesion. Hematoxylin and eosin (H&E) of the skin lesion indicated psoriasiform hyperplasia with compact hyperkeratosis, acanthosis, the lower layers of corneum and epidermis significant parakeratosis, hypogranulosis with vesicular degeneration (**a,b**); Elcetron microscope demonstrated large coarse densely packed bundles of tonofilaments in the keratinocytes of the midmalpighian layer and increased numbers of the coarse keratinohyaline granules in the granular layer. Decreased numbers of chromocytes and pigment granules. The langerhans cells are absent (**c,d**).

The histopathologic findings of plantar skin lesion showed psoriasiform hyperplasia with compact hyperkeratosis, acanthosis, and significant parakeratosis, hypogranulosis with vesicular degeneration on the corneum and epidermis (Fig. 3a,b).

### Ultrastructure features

A biopsy specimen from the plantar skin, 5 months after the initiation of acitretin and salicylate cream, demonstrated large coarse densely packed bundles of tonofilaments in the keratinocytes of the midmalpighian layer and increased numbers of the coarse keratinohyaline granules in the granular layeras well as decreased numbers of chromocytes and pigment granules. The Langerhans cells are absent (Fig. 3c,d).

### Mutation detection

**Figure 4:**
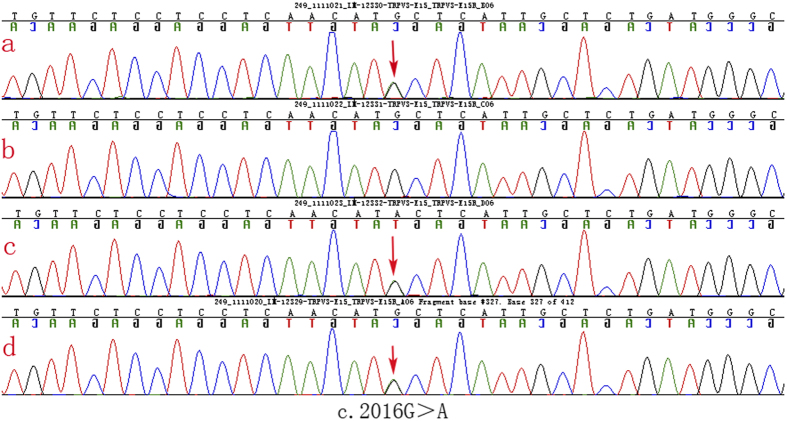
Result of DNA sequencing of *TRPV3*mutation in the Chinese family. *TRPV3* gene sequencing results revealed a identical heterozygous mutation c.2016G > A in the proband, his mother and his maternal grandfather.

We did not detect any pathogenic sequence alterations in *KRT1*, *GJB2*, *LOR* and *SLURP1*. However, *TRPV3* sequencing results revealed an identical heterozygous mutation c.2016G > A in the proband, his mother and his maternal grandfather (Fig. 4a,c,d). Additionally, the mutation wasn’t detected in the proband’s father (Fig. 4b). This mutation is predicted to result in the substitution of an isoleucine for methionine at amino acid position 672 (p.Met672IIe). Meanwhile, this mutation also did not exist in 100 unrelated controls, thus indicating that the mutation was not a single-nucleotide polymorphism. Based on the genetic findings, this familial case was consistent with an autosomal- dominant trait of inheritance.

### Molecular modeling

The conservation score was 9 (Range 1–9; http://conseq.tau.ac.il/). This results suggested Met672 site was highly conserved during evolution and its function may be critical.

**Figure 5:**
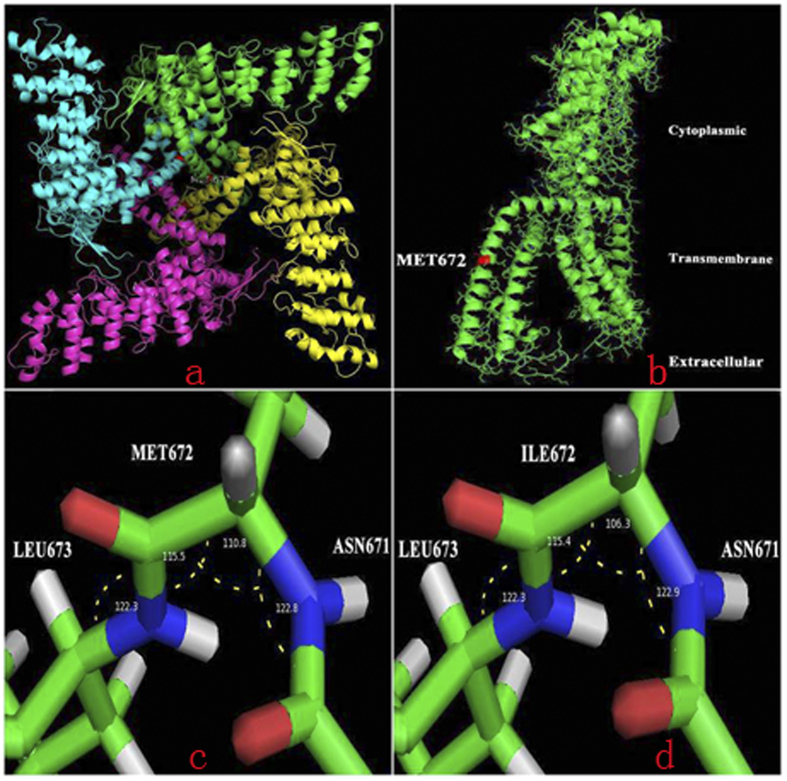
Structural model of the TRPV3 protein. Protein homology modeling found that *TRPV1* could provide ideal templates for homo-tetramer and monomer of *TRPV3* 3D structures prompted that the 672th residue located at a transmembrane domain which may play key roles in the transport of ion channel (**a,b**), the Met672Ile mutation at this site resulted in a change (110.8° to 106.3°) in the angle (**c,d**). And that may ultimately affect the structure and function of *TRPV3*.

Protein homology modeling found that *TRPV1* could provide an ideal template for homo-tetramer and monomer of *TRPV3* 3D structures (Fig. 5a,b) prompted that the 672nd residue located in a transmembrane domain which may play a key role in the transport of ions across the channel. The Met672Ile mutation at this site resulted in a change (110.8° to 106.3°) in the angle (Fig. 5c,d), which ultimately may affect the structure and function of *TRPV3*.

### Genotype-phenotype correlations

To clarify the phenotypic spectrum, we reviewed the clinical features of OS case with known genetic bases, including our familiar case^9^,^10^,^11^,^14^,^15^,^16^,^17^,^18^ (Table 1). To our knowledge, mutations in *TRPV3* have been detected in seven different amino acid residues, including p.Trp521, p.Gly568, p.Gly573, p.Gln580, p.Met672, p.Leu673 and p.Trp692, most of which are autosomal-dominant missense mutations. Exceptionally, the homozygous p.Trp521Ser mutation and heterozygous p.Gly568Cys mutation were described in autosomal-recessive OS cases^10^,^11^.

The Gly573Cys, Gly573Ser and Trp692Gly gain-of-function mutations were identified in six Chinese patients with the full-blown clinical phenotype, including palmplantar keratosis, periorificial keratotic plaques, alopecia, warm-induced pain and itching. The mutation we described was detected in a familial pedigree that presented with minor symptoms and whose clinical traits were partly nonpenetrant. However, a milder phenotype, reported in another Chinese family, was resulted from p.Gln580Pro mutation. Considering that the severity of the disease varied to a large degree between patients with different *TRPV3*mutations, a genotype-phenotype correlation associated with particular amino acid subtitution is likely.

It is worth mentioning that p.Met672Ile and p.Gly568Cys mutation, were associated with distinct phenotype between generations or in one sib pair.

### *In silico* analysis

mCSM and DUET prediction software gave an estimation of the free energy change (ΔΔG), to correlate genotype with phenotype. The predicted values were shown in Table 2. ΔΔG is an energy prediction parameter used to quantitatively compute the protein stability free energy difference, resulting from a single amino acid replacement. It reflects the influence of the mutation upon protein stability due to thermal denaturation.

### CCK8 assay

**Figure 6:**
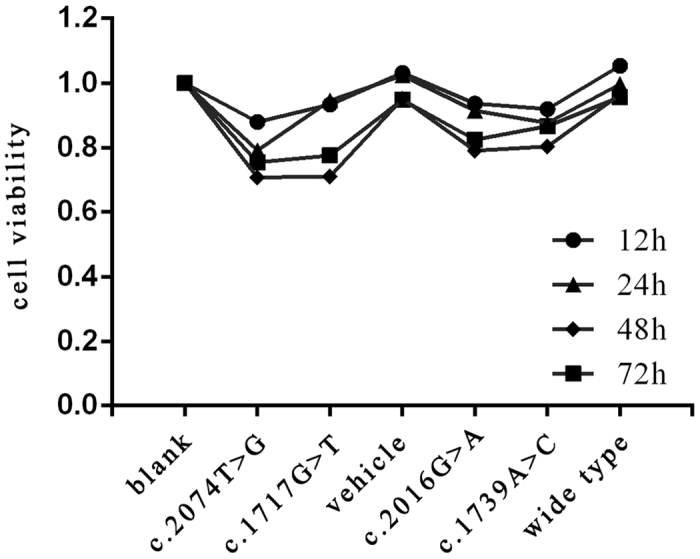
**Induction of cellular apoptosis by mutations in** ***TRPV3***. Cell viability was evaluated by CCK8 after transfection with various mutations in *TRPV3* or wild type TRPV3 gene for 12, 24, 48 and 72 h. Data represent the mean of experiments carried out in triplicate. Statistical analysis for cell viability data was performed using using one-way ANOVA followed by t-test (*P *< 0.05).

To investigate the apoptosis induction effects of four different amino acid substitution on HaCaT cells, cell viability was evaluated by CCK8 after transfection with various mutations in *TRPV3* or wild type TRPV3 gene for 12, 24, 48 and 72 h. As shown in Fig. 6, the four mutations all had apoptosis induction impacts upon HaCaT cell compared with that of the wild type. Cell viability was reduced remarkably after transfection for 48h. The cytotoxicity exerted by G573C and W692G were more obvious, while the cytotoxicity imposed by M672I and Q580P were milder.

### Flow cytometry analysis

**Figure 7:**
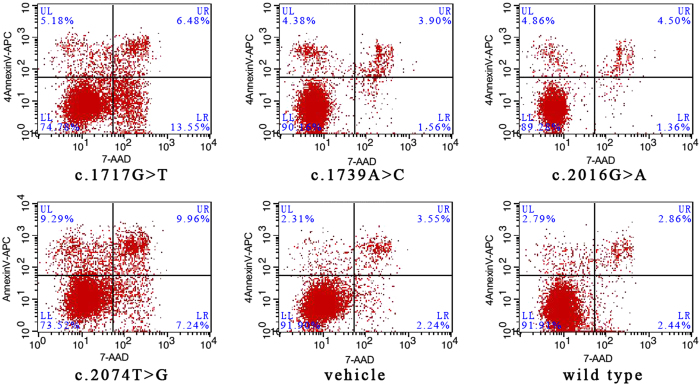
Flow cytometry analysis results. An annexin-V fluorescein APC/7-AAD double stain assay and flow cytometry analysis were performed to confirm cell apoptosis and to explore the differences in the apoptosis induction resulting from these four mutations versus the wild type. The lower left quadrant represents vital cells. The number of early apoptosis cells and late apoptosis cells was indicated in lower right quadrant and upper right quadrant of the histograms, respectively.

An annexin-V fluorescein APC/7-AAD double stain assay and flow cytometry analysis were performed to confirm cell apoptosis and to explore the differences in the apoptosis induction resulting from these four mutations versus the wild type. The lower left quadrant represents vital cells. The number of early apoptosis cells and late apoptosis cells was indicated in lower right quadrant and upper right quadrant of the histograms, respectively. As shown in Fig. 7, transfection with p.Gly573Cys, p.Trp692Gly, p.Met672IIe and p.Gln580Pro increased the number of apoptosis cells in varying degrees compared with the wild type. In contrast, p.Gly573Cys and p.Trp692Gly exerted more significant induction of apoptosis upon HaCaT cells. The degree of the induction of apoptosis was consistent with the phenotype resulting from the corresponding mutation, which might hint towards the underlying mechanism mediating the genotype-phenotype correlation.

## Discussion

OS is a genetically heterogeneous keratinization disorder characterized by diffuse, transgredient PPK resulting in flexural deformities and spontaneous amputation of the fingers or toes, usually accompanied by periorificial keratosis. Meanwhile, a minority of published cases have described rare, atypical clinical features. To the best of our knowledge, none of the previously reported OS cases had clinical signs overlapping with Huriez syndrome. Here we presented an atypical familial case of OS sharing some clinical features with Huriez syndrome, namely, a scleodactyly-like appearance and cone-shaped hands. Huriez syndrome is also a rare autosomal dominant keratinization disorder characterized by PPK, diffuse scleroatrophy of the hands with sclerodactyly, tapered fingers, hypohidrosis and hypoplastic nails^19^. Squamous cell carcinoma is usually associated with Huriez syndrome^20^. Ultrastructural assay revealed an absence of Langerhans cells in the affected skin, which might lead to the tendency of skin lesions to undergo malignant changes^21^. Although our case had some clinical signs previously described in Huriez syndrome, the resemblance was not sufficient to make a conclusive diagnosis. To determine whether the current case was actually Huriez syndrome, pathological and ultrastructural examinations of the proband’s skin lesions were routinely performed. Light microscopy revealed orthokeratotic hyperkeratosis with parakeratosis, hypogranulosis and psoriasiform hyperplasia. Similar nonspecific features have been described not only in Huriez syndrome, but also in different kinds of PPK, such as Olmsted syndrome and Vohwinkel syndrome^3^,^22^,^23^. Therefore, the histopathologic finding was inconclusive. Ultrastructural assay further revealed depletion of Langerhans cells in the affected skin, which resulted in difficulty in ruling out Huriez syndrome. In fact, the absence or reduction of Langerhans cells merely indicate the susceptibility of skin neoplasms. Except for Huriez syndrome, several other cornification disorders such as OS and Keratitis-Ichthyosis-Deafness (KID) syndrome, have also been associated with an increased vulnerability of squamous cell carcinoma^7^,^24^. Therefore, the reduction or deletion of Langerhans cells in involved skin may not be unique to Huriez syndrome. In view of this, we could not definitively diagnose this familial case based solely on the pathological or ultrastructural findings or clinical features.

Apart from the scleodactyly-like appearance and tapered fingers, pseudoainhum was also a remarkable clinical sign in the present case. In an attempt to acquire a definitive diagnosis, we screened five genes closely correlated with mutilating PPK. As a result, we identified a heterozygous guanine-to-adenine transition at position 2016 within *TRPV3*. We further ruled out the possibility of a single-nucleotide polymorphism by sequencing *TRPV3* in 100 normal individuals. The mutation was also absent from the 1000 Genomes Project database and dbSNP. To evaluate the effect of the Met672Ile mutation, we performed a comprehensive analysis on the structural change of the TRPV3 protein. Initially, the mutation affected a highly conserved residue. Next, we simulated the three-dimensional (3D) structure of TRPV3 protein. This analysis revealed that the Met672Ile mutation may ultimately affect the structure and function of TRPV3 protein. Additionally, a functional assay indicated that the Met672Ile mutation imposed a more significant induction of apoptosis on HaCaT cells compared with that of the wild type. Despite the fact that this mutation has not yet been reported, above-mentioned facts support the possibility that it is pathogenic. Meanwhile, considering the presence of pseudoainhum and erythromelalgia in the proband, we made a final diagnosis of atypical OS. The scleodactyly-like appearance and tapered fingers may represent an unreported phenotype of OS. Recently, *TRPV3* has been associated with skin inflammation and wound healing, prompting the aberrant activation of the *TRPV3* ion channel might have potential relevance to the phenotype of scleodactyly and tapered fingers^25^,^26^.

*TRPV3* has a significant role in mediating itch and pain sensation, regulating physiological skin homeostasis, involving skin inflammation, wound healing^26^,^27^, and modulating hair growth^28^, which indicate that OS could encompass a wide range of clinical manifestations. Therefore, the functional impact of different point mutations on the the transmembrane protein could contribute to distinct phenotypes. To better understand the genotype-phenotype correlation, we further combined *in silico* predictions with functional assays, which linked the site of the mutation in *TRPV3* with the clinical outcome.

Initially, the ΔΔG of the corresponding mutations was quantitatively computed, and it served as a quantitative index for the influence of each mutation on the stability of TRPV3 protein (Table 2). A positive value indicates that the energy will be lower as the mutagenesis. Based on this premise, the mutation will contribute to a relatively stable protein structure, which may correspond to a mild phenotype. Instead, the negative value indicates that the energy will be higher, thus corresponding to a destabilized protein structure. To some extent, the greater the change in the absolute value of the energy, the more the function of the ion channel will be influenced. Therefore, more serious symptoms could manifest due to an ion channel that had a greater conformational change. The only positive value was due to the Gln580Pro mutation (Table 2), which resulted in a relatively stable protein structure and caused the mildest phenotypic appearance. On the other hand, ΔΔG value resulting from other eight point mutations in *TRPV3* were all negative values that were associated with less stable protein structures and more severe phenotypes. Among the eight mutations, the absolute energy change resulting from the Met672Ile mutation in our case was less than the other seven mutations, which in turn caused a less dramatic conformational change on the ion channel and a relatively moderate clinical manifestation. In fact, the penetrance of phenotype resulting from Met672Ile exactly fell in between that of Gln580Pro and other mutations (Table 1).

Nevertheless, it should be emphasized that *in silico* predictions can only provide an approximation for pathogenicity and cannot completely substitute for a functional assays. Thus, we further performed experimental validation to functionally assess the effects of mutations in *TRPV3*, hopefully preventing mistakens due to *in silico* estimations alone. We mutagenized four highly conserved residues (G573, Q580, M672 and W692) to test whether the functional impacts were dependent on the site of amino acid exchange. Interestingly, a CCK8 assay revealed G573C and W692G had more significant apoptosis induction effects on HaCaT cells compared with those of M672I and Q580P. Flow cytometry analysis further demonstrated G573C, W692G more remarkably increased the number of the apoptosis cells, whereas Q580P slightly enhanced the rate of apoptosis. In contrast, the impact on apoptosis exerted by M672I fell in between that of G573C, W692G and Q580P. Overall, the *in silico* estimation and functional data consistently corroborated the notion of a genotype-phenotype relationship being associated with the specific site of the mutation in *TRPV3*. However, an intra-family phenotypic discrepancy was reported in patients with an identical mutation in two cases, which indicated the phenotypic appearance maybe influenced by other factors such as modifier genes, environmental aspect and immunoregulation.

Overall, the present study identified a novel mutation in *TRPV3* in an atypical familial case, thus expanding and updating the mutational, phenotypic spectrum of OS. Additionally, we further elucidated the genotype-phenotype correlation by combining *in vitro* and *in silico* datasets.

## Method

### Patient Recruitment

All clinical investigations have been conducted according to the principles expressed in the Declaration of Helsinski. This study was approved by the Ethics Committee of ShangHai JiaoTong University School of Medicine. Written informed consent was obtained from each participant. If the participants are younger than 18 years old, written informed consents were signed by the parents on behalf of the children. A 2ml venous blood sample was drawn into an ethylenediamine tetraacetic acid (EDTA) sample tube. Genomic DNA was extracted from peripheral blood leukocytes using the standard phenol/chloroform extraction protocols.

### Mutation Detection

We designed primers flanking all coding exons and intron-exon boundaries of the *KRT1*, *GJB2*, *LOR*, *SLURP1* and *TRPV3* using the web-based version of the Primer 3.0 program (http://www.genome.wi.mit.edu./cgi-bin/primer/prime 3_www.cgi) . PCR was performed in 15 μl reaction volume containing 20 ng of genomic DNA,0.3 mM dNTPs, 0.3 μM of each primer, 0.3 mM Mgcl_2_ and 0.1 U of Taq DNA polymerase. The PCR condition were: Taq activation at 95 °C for 15min,followed by 40 cycles, each having denaturation at 94 °C for 40s, annealing at 58 °C for 60s, and extension at 72 °C for 55s, except that in the first ten cycles the annealing temperature decreased from 63 °C to 58 °C by 0.5 °C per cycle, and the final extension was 72 °C for 10 min. After the amplification, the products were purified using a QIA quick PCR Purification Kit (Qiagen). We sequenced the five genes using the ABI PRISM®3730 automated sequencer(Applied Biosystems).Sequence analysis was performed in both orientations.

### Histopathological observation

Following overnight fixation in 4% paraformaldehyde, the plantar skin lesion were dehydrated by stepwise transfer into increasing concentrations of ethanol and embedded in paraffin. Embeded tissues were sliced into 4 μm sections using a Leica automatic microtome and stained with hematoxylin and eosin (H&E) for histological assessment under light microscopy.

### Transmission electron microscopy examination

Plantar skin lesion were fixed in 2.5% glutaraldehyde in 0.1 M phosphate buffer (PH 7.4) at 4 °C for 24 hr. Samples were then washed twice with phosphate buffer (0.1 M, PH 7.4) and post-fixed for 20 min with 1% osmium tetroxide in 0.1 M phosphate buffer (PH 7.4). After dehydrated, samples were embedded in Epon-812 epoxy resin and ultrathin sections were made using a LKB-I ultramicrotome (LKB,Bromma, Sweden). Sections were mounted on copper grids and stained with 2% uranyl acetate in a 1% solution of lead citrate for 30min. The ultrastructure of the skin lesion was visualized using a PHILIPS CM-120 transmission electron microscopy operating at 200 kV. Sections were photographed with a Gatan 832 CCD camera.

### Bioinformatic analysis

Online tools: ConSurf^29^ were used to predict conservation score. And then, protein homology modeling was conducted by Swiss-model sever to construct the 3D structure of *TRPV3*^30^. Transient receptor potential cation channel subfamily V member 1 (gene name: *TRPV1*; PDB entry: 3J5P; sequence identity with *TRPV3*: 46%) with 6 transmembrane α-helixes was the appropriate structure model^31^. RosettaBackrub sever provided the protein structure with Met672Ile point mutation^32^. PyMOL (Schrödinger, version 1.3) displayed and optimized the protein structure. At last, to systematically estimate the change of *TRPV3* protein upon mutations (Trp521Ser, Gly568Cys, Gly573Cys, Gly573Ser, Gly573Ala, Gln580Pro, Met672Ile, Leu673Phe, Trp692Gly, Trp692Cys), mCSM and DUET web server was performed quantitatively compute the free energy change (ΔΔG)^33^,^34^.

### Cell culture, vectors and transfection

HaCaT cells were cultured in DMEM medium (Gibco, Grand Island, NY), supplemented with 10% fetal bovine serum (Gibco). Cells were incubated in an incubator containing 5% CO2 at 37 °C . Cells were passaged when cellular confluence reached 70% or more. Cells were harvested at the logarithmic phase for use.

*TRPV3* expression vector pLV.Ex3d.P/puro-TRPV3 was constructed by replacement of the GFP fragment of the pLV.Ex3d.P/puro vector (Cyagen) with the TRPV3 (RefSeq NM_001258205.1) coding sequence amplified from the plasmid. Mutants were generated via site-directed mutagenesis.

The cells were transiently transfected using Lipofectamine 2000 , according to the manufacturer’s protocol (Invitrogen).

### CCK8 assay

The Cell Count Kit-8 (CCK8, Dojindo, Rockville, MD, USA) was used to assess the effects of different mutations on cell viability. The cells were divided into 7 groups: blank, mock-vehicle, wild type, Q580P, M672I, G573C and W692G— and seeded in a 96-well plate at a density of 1,000 cells per well; every group has 3 wells. The CCK8 kit was used to detect the apoptosis of HaCaT cells 12, 24, 48 and 72 hours after seeding. Subsequently the cell viability was evaluated by CCK8 following the manufacturer’s instructions. The absorbance at wavelength 450 nm was measured for the supernatant of each well using the plate reader Multiskan EX (Thermo Fisher Scientific Inc., Waltham, MA, USA). The experiment was performed in triplicate.

### Flow cytometry detection

Apoptosis was determined by flow cytometry analysis. HaCaT cells were collected after transiently transfected with or without mutation for 48h. Annexin-V APC/7-AAD double stain assays (Yeasen Inc, Shanghai, China) were performed following the manufacturer’s protocol. Both floating and trypsinized adherent cells were collected, resuspended in 500 μL of binding buffer containing 5 μL of annexin-V APC and 5 μL of 7-AAD, and then incubated for 10 min in the dark at room temperature before flow cytometry analysis.

### Statistical analysis

Values were expressed as means ± SD (standard deviation). One-way analysis of variance (ANOVA) followed by t-test was used for statistical analysis. Probability (*p*) values less than 0.05 were considered significant.

## Additional Information

**How to cite this article**: Ni, C. *et al.* A novel mutation in TRPV3 gene causes atypical familial Olmsted syndrome. *Sci. Rep.* **6**, 21815; doi: 10.1038/srep21815 (2016).

## Figures and Tables

**Table 1:** Clinical features and *TRPV3* genotypes of OS patients.

Origin	Sex	Clinical features	Family history	Genotype
China	female	Palmoplantar keratosis, constricting digit bands, periorificial keratosis, alopecia, warm-induced pain and itching.	−	c.1717G > A/Wild type
China	female	Palmoplantar keratosis, periorificial keratosis, alopecia,warm- induced pain and itching.	−	c.1717G > T/Wild type
China	female	Palmoplantar keratosis, spontaneous digit amputation, periorificial keratosis, alopecia, warm-induced pain and itching.	+	c.1717G > A/Wild type
China	female	Palmoplantar keratosis, constricting digit bands, spontaneous digit amputation, periorificial keratosis, alopecia, warm-induced pain and itching.	−	c.1717G > A/Wild type
China	female	Palmoplantar keratosis, constricting digit bands, periorificial keratosis, dry curly hair ,warm- induced pain and itching.	−	c.2074T > G/Wild type
China	male	Palmoplantar keratosis, constricting digit bands, periorificial keratosis, alopecia, warm-induced pain and itching.	−	c.1717G > A/Wild type
Iran	male	Palmoplantar keratosis, constricting digit bands, periorificial keratosis, alopecia, warm-induced pain, sparse eyelashes and eyebrows	−	c.2076G > C/Wild type
Caucasia	male	Palmoplantar keratosis, constricting digit bands, periorificial keratosis, warm-induced pain and itch, frequent bacterial and fungal infections of the skin.	−	c.1718 G > C/Wild type
India	male	Palmoplantar keratosis, spontaneous digit amputation, periorificial keratosis, warm-induced pain, dry curly hair,dystrophyic nails.	−	c.1717G > A/Wild type
Israel	male	Palmoplantar keratosis, spontaneous digit amputation, periorificial keratosis, warm-induced pain	−	c.1562 G > C /c.1562 G > C
France	male	Palmoplantar keratosis, warm-induced pain, fine, curly and dry hair, thin and brittle nails.	−	c. 2017 C > T/Wild type
France	male	severe plantar keratoderma associated with intense erythromelalgia, itching.	+	c. 1702 G >T/ c.784 + 1G>A.
France	male	Moderate and focal plantar keratoderma, mild erythromelalgia, xerosis, eczema of the ears and blepharitis associated with the loss of his eyelashes.	+	c. 1702 G > T/c.784 + 1G > A
China	male	Focal palmoplantar keratosis, mild erythromelalgia	+	c. 2016 G > A/Wild type
China	female	Focal palmoplantar keratosis with obvious pseudoainhum	+	c. 2016 G > A/Wild type
China	male	Focal plantar keratoderma, desquamation on the palms	+	c. 2016 G > A/Wild type
China	male	Focal plantar keratoderma	+	c. 1739 A > C/Wild type
China	male	Focal plantar keratoderma	+	c. 1739 A > C/Wild type

**Table 2:** The relation between the free energy change(ΔΔG) and the predicted phenotype.

Protein variant	Genotype	mCSM Predicted Stability Change (ΔΔG)	DUET Predicted Stability Change (ΔΔG)	Predicted phenotype
Q580P	c. 1739 A > C/Wild type	0.365 Kcal/mol (*Stabilizing*)	0.129 Kcal/mol (*Stabilizing*)	mild
M672I	c. 2016 G > A/Wild type	−0.627 Kcal/mol (Destabilizing)	−0.209 Kcal/mol (Destabilizing)	medium
G573C	c. 1717 G > T/Wild type	−1.549 Kcal/mol (Destabilizing)	−1.371 Kcal/mol (Destabilizing)	severe
G573S	c. 1717G > A/Wild type	−1.794 Kcal/mol (*Destabilizing*)	−1.672 Kcal/mol (*Destabilizing*)	severe
G573A	c. 1718 G > C/Wild type	−0.96 Kcal/mol (*Destabilizing*)	−0.674 Kcal/mol (*Destabilizing*)	severe
L673P	c. 2017 C > T/Wild type	−1.429 Kcal/mol (*Destabilizing*)	−1.613 Kcal/mol (*Destabilizing*)	severe
W692G	c. 2074T > G/Wild type	−4.587 Kcal/mol (*Destabilizing*)	−4.059 Kcal/mol (*Destabilizing*)	severe
W692C	c. 2076G > C/Wild type	−2.701 Kcal/mol (Destabilizing)	−2.323 Kcal/mol (Destabilizing)	severe
W521S	c.1562 G > C/c. 1562 G > C	−2.192 Kcal/mol (*Destabilizing*)	−1.895 Kcal/mol (*Destabilizing*)	severe

## References

[b1] Atherton D. J., Sutton C. & Jones B. M. Mutilating palmoplantar keratoderma with periorificial keratotic plaques (Olmsted’s syndrome). Br J Dermatol 122, 245–52 (1990). 2138494 10.1111/j.1365-2133.1990.tb08271.x

[b2] Frias I. J. *et al.* Olmsted syndrome: report of a new case. Br J Dermatol 136, 935–8 (1997). 9217830

[b3] Ahmad N. *et al.* Nonmutilating palmoplantar and periorificial keratoderma: a variant of Olmsted syndrome or a distinct entity? International Journal of Dermatology 49, 658–665 (2010). 20618471 10.1111/j.1365-4632.2009.04429.x

[b4] Judge M. R., Misch K., Wright P. & Harper J. I. Palmoplantar and periorificial keratoderma with corneal epithelial dysplasia: a new syndrome. Br J Dermatol 125, 186–188 (1991). 1832929 10.1111/j.1365-2133.1991.tb06070.x

[b5] Mevorah B. *et al.* Olmsted syndrome: Mutilating palmoplantar keratoderma with periorificial keratotic plaques. J Am Acad Dermatol 53, s266–72 (2005). 16227106 10.1016/j.jaad.2005.03.036

[b6] Requena L. *et al.* Olmsted syndrome: Report of a Case With Study of the Cellular Proliferation in Keratoderma. The American Journal of Dermatopathology 23, 514–520 (2001). 11801792 10.1097/00000372-200112000-00003

[b7] Tao J. *et al.* Olmsted syndrome: a case report and review of literature. International Journal of Dermatology 47, 432–437 (2008). 18412857 10.1111/j.1365-4632.2008.03595.x

[b8] Duchatelet S. & Hovnanian A. Olmsted syndrome: clinical, molecular and therapeutic aspects. Orphanet Journal of Rare Diseases 13, 015–024 (2015). 10.1186/s13023-015-0246-5 PMC4373112 25886873

[b9] Lin Z. M. *et al.* Exome sequencing reveals mutations in TRPV3 as a cause of Olmsted syndrome . Am J Hum Genet. 90, 558–564 (2012). 22405088 10.1016/j.ajhg.2012.02.006 PMC3309189

[b10] Eytan O. *et al.* Olmsted Syndrome Caused by a Homozygous Recessive Mutation in TRPV3. J Invest Dermatol 134, 1752–4 (2014). 24463422 10.1038/jid.2014.37

[b11] Duchatelet S. *et al.* Olmsted syndrome with erythromelalgia caused by recessive transient receptor potential vanilloid 3 mutations. Br J Dermatol 171, 675–8 (2014). 24606194 10.1111/bjd.12951

[b12] Haghighi A. *et al.* A missense mutation in the MBTPS2 gene underlies the X-linked form of Olmsted syndrome. J Invest Dermatol 133, 571–3 (2012). 22931912 10.1038/jid.2012.289

[b13] Wang H. J. *et al.* Recurrent splice-site mutation in MBTPS2 underlying IFAP syndrome with Olmsted syndrome-like features in a Chinese patient. Clin Exp Dermatol. 39, 158–61 (2014). 24313295 10.1111/ced.12248

[b14] Lai-Cheong J. E. *et al.* Recurrent heterozygous missense mutation, p. Gly573Ser,in the *TRPV3* gene in an Indian boy with sporadic Olmsted syndrome. Br J Dermatol 167, 440–2 (2012). 22835024 10.1111/j.1365-2133.2012.11115.x

[b15] Danso-Abeam D. *et al.* Olmsted syndrome: exploration of the immunological phenotype. Orphanet J Rare Dis 79, 8 (2013). 10.1186/1750-1172-8-79 PMC3662572 23692804

[b16] Kariminejad A., Barzegar M., Abdollahimajd F., Pramamik R. & McGrath J. A. Olmsted syndrome in an Iranian boy with a new *de novo* mutation in *TRPV3*. Clin Exp Dermatol 39, 492–495 (2014). 24758389 10.1111/ced.12318

[b17] Duchatelet S. *et al.* A new TRPV3 missense mutation in a Patient With Olmsted Syndrome and Erythromelalgia. JAMA Dermatol 150, 303–6 (2014). 24452206 10.1001/jamadermatol.2013.8709

[b18] He Y. *et al.* A Gain-of-Function Mutation in TRPV3 Cause Focal Palmoplantar Keratoderma in a Chinese Family. J Invest Dermatol doi: 10.1038 (2014). 10.1038/jid.2014.429 25285920

[b19] Kavanagh G. M., Jardine P. E., Peachey R. D., Murray J. C. & De-Berker D. The scleroatrophic syndrome of Huriez. Br J Dermatol 137, 114–118 (1997). 9274637

[b20] Sekar S. C. & Srinivas C. R. Huriez syndrome. Indian J Dermatol Venereol Leprol 74, 409–410 (2008). 18797084 10.4103/0378-6323.42930

[b21] Guerriero C. *et al.* Huriez syndrome: case report with a detailed analysis of skin dendritic cell. Br J Dermatol 143, 1091–6 (2000). 11069529 10.1046/j.1365-2133.2000.03793.x

[b22] Watanabe E., Taki T., Ichihashi M. & Ueda M. A. Nonfamilial Japanese Case of Huriez Syndrome: p53 Expression in Squamous Cell Carcinoma. Dermatology 207, 82–84 (2003). 12835558 10.1159/000070951

[b23] Corte L. D. *et al.* Vohwinkel syndrome: ichthyosiform variant-by camisa-case report. An Bras Dermatol 88, 206–8 (2013). 24346921 10.1590/abd1806-4841.20132135 PMC3875996

[b24] Kimyai-Asadi A., Kotcher L. B. & Jih M. H. The molecular basis of hereditary palmoplantar kerato- dermas. J Am Acad Dermatol 47, 327–43 (2002). 12196741 10.1067/mjd.2002.124814

[b25] Steinhoff M. & Tamás Bíró. A TR(I)P to Pruritus Research Role of TRPV3 in Inflammation and Itch. J Invest Dermatol 129, 531–535 (2009). 19209153 10.1038/jid.2008.440

[b26] Yang P. & Zhu M. X. TRPV3. Handb Exp Pharmacol 222, 273–91 (2014). 24756710 10.1007/978-3-642-54215-2_11

[b27] Nilius B., Bíró T. & Owsianik G. TRPV3:time to decipher a poorly understood family member! 592, 295–304 (2014). 10.1113/jphysiol.2013.255968 PMC3922494 23836684

[b28] Borbíró I. *et al.* Activation of Transient Receptor Potential Vanilloid-3 Inhibits Human Hair Growth. J Invest Dermatol 131, 1605–14 (2011). 21593771 10.1038/jid.2011.122

[b29] Ashkenazy H., Erez E., Martz E., Pupko T. & Ben-Tal N. Consurf 2010: Calculating evolutionary Conservation in sequence and structure of proteins and nucleic acids. Nucleic Acids Res 38, 529–33 (2010). 10.1093/nar/gkq399 PMC2896094 20478830

[b30] Biasini M. *et al.* SWISS-MODEL: modeling protein tertiary and quaternary structure using evolutionary information. Nucleic Acids Res 42, 252–8 (2014). 10.1093/nar/gku340 PMC4086089 24782522

[b31] Liao M., Cao F., Julius D. & Cheng Y. Structure of the TRPV1 ion channel determined by electron cryomicroscopy. Nature 504, 107–12 (2013). 24305160 10.1038/nature12822 PMC4078027

[b32] Lauck F., Smith C. A., Friedland G. F., Humphris E. L. & Kortemme T. RosettaBackruba web server for flexible backbone protein structure modeling and design. Nucleic Acids Res 38, 569–75 (2010). 10.1093/nar/gkq369 PMC2896185 20462859

[b33] Pires D. E., Ascher D. B. & Blundell T. L. Mcsm: predicting the effects of mutations in proteins using graph-based signatures. Bioinformatics 30, 335–42 (2014). 24281696 10.1093/bioinformatics/btt691 PMC3904523

[b34] Pires D. E., Ascher D. B. & Blundell T. L. DUET: a server for predicting effects of mutations on protein stability using an integrated computational approach. Nucleic Acids Res 42, 314–9 (2014). 10.1093/nar/gku411 PMC4086143 24829462

